# An Enhanced Q-Factor Cantilever Resonator in Viscous Liquids Using Strategic Perforation †

**DOI:** 10.3390/mi17030385

**Published:** 2026-03-22

**Authors:** Song Qu, Cao Xia

**Affiliations:** 1 School of Mechanical and Aerospace Engineering, Jilin University, Changchun 130022, China; 2 School of Mechanical Engineering, Sichuan University, Chengdu 610106, China

**Keywords:** cantilever resonators, fluid damping, signal-to-noise ratio, perforation approach, Q-factor, structure stiffness

## Abstract

Cantilever resonators immersed in liquids experience significant viscous damping, which degrades the resonator’s quality factor (Q-factor) and lowers the signal-to-noise ratio. To address this challenge, a strategic perforation approach is proposed to enhance the Q-factor of cantilever resonators in viscous liquids. A distributed-parameter model based on the Rayleigh–Ritz method is developed to quantify the spatial distribution of structural stiffness and viscous damping. The analysis shows that material removal at the free end effectively reduces squeeze-film damping while maintaining stiffness. Resonator prototypes with different perforation designs are fabricated and tested in various viscous liquids. The results show that the free-end perforated cantilever (FPC) achieves a higher Q-factor compared to the conventional non-perforated cantilever (NPC). In an 18.5 mPa·s liquid, the FPC demonstrates a 346.2 % Q-factor enhancement and a 4.78 % frequency increase. These results provide a design guideline for high-performance cantilever resonators in liquid-phase sensing applications.

## 1. Introduction

Cantilever resonators are widely used in biomedical diagnostics [1,2,3], petrochemical monitoring [4,5,6], atomic force microscope (AFM) probes [7,8,9], and resonant sensing technology [10,11,,12] due to their small size [13,14], fast response [15,16], and compatibility with microelectromechanical systems (MEMSs) [17,18]. When operated in gaseous or vacuum environments, these resonators exhibit a high quality factor (Q-factor) [7,19], enabling precise frequency resolution. However, once immersed in liquids, they experience significant viscous damping arising from fluid viscosity. This damping manifests as squeeze-film effects and added mass loading, which accelerate energy dissipation and substantially degrade the Q-factor [20]. The resulting reduction in Q-factor directly lowers the signal-to-noise ratio and broadens the resonance peak, making accurate extraction of resonant frequency challenging. Consequently, the performance of cantilever-based devices in liquid environments suffers from limited sensitivity and reliability, restricting their full potential in liquid-phase applications.

Enhancing the Q-factor of cantilever resonators in viscous liquids is therefore a critical step toward overcoming this fundamental limitation. Various strategies have been explored to address this challenge. For instance, dual-resonant-frequency models have been proposed to decouple liquid properties from the measured response, avoiding direct dependence on the Q-factor [21,22]. However, these models do not mitigate the underlying amplitude attenuation, leaving the resonance peaks flattened and difficult to resolve in highly viscous media. Zhao et al. [23] investigated the use of higher-order resonant modes, which exhibit higher equivalent dynamic stiffness against fluid loading. While this approach improves the Q-factor to some extent, exciting higher-order modes in liquids demands substantially higher actuation energy, increasing power consumption and complicating the driving electronics. Partially immersed cantilever configurations have also been reported [24,25,26], which reduce fluid contact area to alleviate damping. Nevertheless, these designs are challenging to integrate with standard MEMS fabrication processes, and the air-liquid interface introduces unpredictable surface tension effects that further degrade the signal-to-noise ratio. Despite these efforts, existing methods either fail to address the root cause of viscous damping or introduce new complexities that limit practical applicability.

To overcome the limitations of existing strategies, this article proposes free-end perforation to enhance the Q-factor of cantilever resonators in viscous liquids. Unlike dual-frequency models that analytically decouple liquid properties without reducing physical damping, or higher-order modes that require high actuation energy, our approach alters the fluid–structure interaction in the fundamental mode. Introducing a perforation at the free end creates a vertical escape path for the trapped fluid. This mechanism shortens the flow path and suppresses squeeze-film damping while preserving structural stiffness. Consequently, this passive design realizes high Q-factor cantilever resonators in liquids without the need for complex driving electronics or partial immersion.

The contributions of this article are as follows:

(1) A passive free-end perforation approach is developed to suppress squeeze-film damping and enhance the Q-factor of cantilever resonators.
(2) A distributed-parameter model is derived to map the damping distribution and structural stiffness for designing resonators with maximum fluid damping suppression.
(3) Cantilever resonators with different perforation designs are fabricated and tested in various viscous liquids, validating the proposed approach.

## 2. Physical Mechanism and Theoretical Model

### 2.1. Physical Mechanism of the Proposed Approach

**Figure 1:**
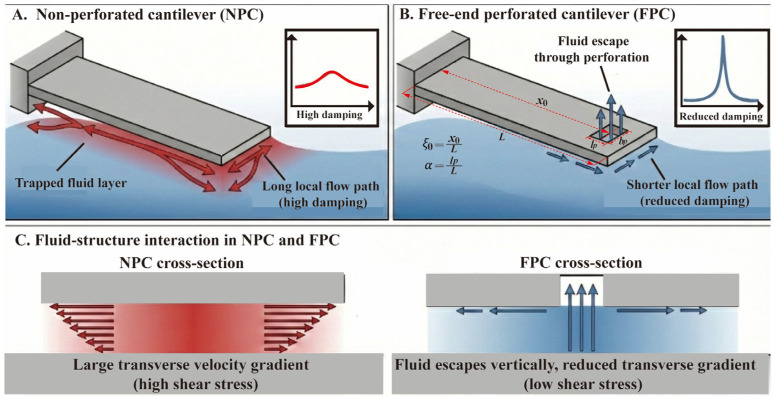
Schematic illustration of the Q-factor enhancement mechanism: (**A**) Non-perforated cantilever (NPC); (**B**) Free-end perforated cantilever (FPC); (**C**) Fluid–structure interaction mechanism of the NPC and FPC. The normalized perforation length is denoted by α, and the normalized perforation position is denoted by $\xi_{0}$. The arrows indicate the main fluid escape directions and flow paths. The color gradient qualitatively represents the relative viscous damping or shear effect, with red for stronger and blue for weaker effects.

Figure 1 illustrates the Q-factor enhancement mechanism of the proposed microcantilever resonator, which suppresses squeeze-film damping by introducing a perforation near the free end. The underlying principle is that the trapped fluid escapes through the perforation rather than flowing only along the lateral direction. This shortens the fluid escape path, reduces the transverse velocity gradient, and suppresses squeeze-film damping. In the present work, the reduction in viscous damping is the main mechanism for Q-factor enhancement, while the effects of stiffness and effective mass changes are accounted for in the theoretical analysis in Section 2.3 and Section 2.4.

### 2.2. Theoretical Model of the Stiffness and Damping Distribution

For a cantilever vibrating in an incompressible viscous fluid, the transverse motion is governed by the Euler–Bernoulli beam equation:

(1) $$I\frac{\partial^{2}w\left(x,t\right)}{\partial x^{4}}+\rho_{b}A\frac{\partial^{2}w\left(x,t\right)}{\partial t^{2}}=F_{hydro}\left(x,t\right)+F_{ext}\left(x,t\right)$$

where E is Young’s modulus, I is the moment of inertia, $\rho_{b}$ and A are the beam density and cross-sectional area, $w\left(x,t\right)$ is the transverse displacement, and $F_{ext}\left(x,t\right)$ is the external excitation. Following Sader’s formulation [27], the hydrodynamic load per unit length is $F_{hydro}\left(x,t\right)=\frac{\pi }{4}\rho_{f}b^{2}\omega^{2}\Gamma \left(\omega \right)w\left(x,t\right)$, where $\rho_{f}$ is the fluid density, b is the beam width, ω is the angular frequency, and $\Gamma \left(\omega \right)$ is the complex hydrodynamic function.

Using separation of variables, $w\left(x,t\right)=q(t)\phi \left(x\right)$, and introducing the dimensionless coordinate ξ=x/L, the fundamental mode shape for a clamped-free cantilever is

(2) $$\begin{array}{c} \phi \left(\xi \right)=\left(cosh\beta \xi -cos\beta \xi \right)-\sigma \left(sinh\beta \xi -sin\beta \xi \right)\; \end{array}$$

with eigenvalue β=1.8751 and constant σ = 0.7341.

The effective stiffness $K_{eff}$ is obtained by equating the strain energy of the continuous beam to that of an equivalent single-degree-of-freedom system:

(3) $$K_{eff}=\frac{EI}{L^{3}}\int_{0}^{1}{\left(\phi^{\prime \prime }\left(\xi \right)\right)}^{2}d\xi =\frac{EI}{L^{3}}I_{K}$$

where $I_{K}$ is the stiffness integration constant. For the fundamental mode, $I_{K}=12.362$.

The equivalent damping $C_{eff}$ is obtained by equating the viscous power dissipation of the continuous beam to that of the equivalent single-degree-of-freedom system:

(4) $$C_{eff}=c_{f}L\int_{0}^{1}{\left(\phi \left(\xi \right)\right)}^{2}d\xi =c_{f}LI_{C}\;$$

where $c_{f}$ is the damping per unit length and $I_{C}$ is the damping integration constant. For the fundamental mode, $I_{C}=1$.

**Figure 2:**
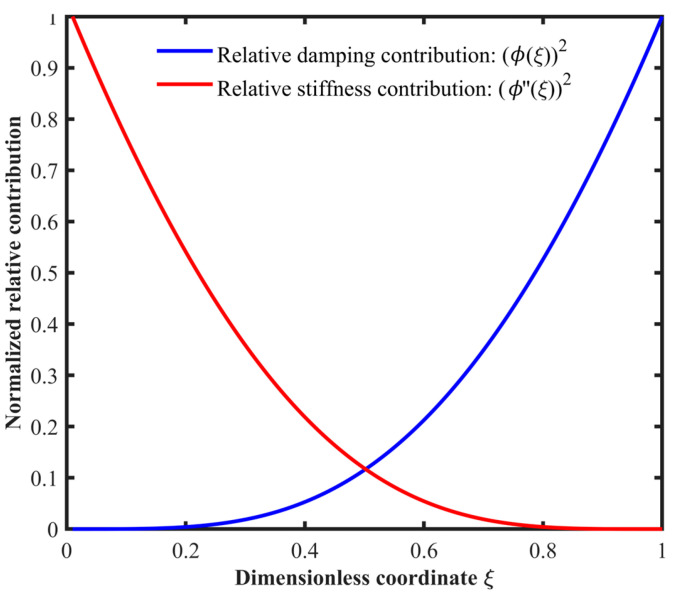
Distribution of damping weighting function $(\phi (\xi ){)}^{2}$ and stiffness weighting function $(\phi^{\prime \prime }(\xi ){)}^{2}$ along the normalized cantilever length.

Equations (3) and (4) indicate that the equivalent stiffness and damping are weighted by ${\left(\phi^{\prime \prime }\left(\xi \right)\right)}^{2}$ and ${\left(\phi \left(\xi \right)\right)}^{2}$, respectively. These weighting functions describe how local material removal at different longitudinal locations affects the equivalent stiffness and damping. Figure 2 shows their distributions along the beam. The stiffness weighting function ${\left(\phi^{\prime \prime }\left(\xi \right)\right)}^{2}$ is concentrated near the clamped end and decreases toward the free end, whereas the damping weighting function ${\left(\phi \left(\xi \right)\right)}^{2}$ increases toward the free end.

### 2.3. Theoretical Analysis of Perforation Location on Stiffness and Damping

In the theoretical analysis, the perforation is described by two variables: the normalized perforation position $\xi_{0}=x_{0}/L$ and the normalized perforation length $\alpha =l_{p}/L$, as illustrated in Figure 1. The perforation width $b_{p}$ is fixed and is not treated as an independent variable in the present model. The free-end perforation considered here is a local rectangular hole near the beam tip rather than a full-width truncation of the beam. Therefore, the total beam length remains L.

Equations (5) and (6) are derived from a reduced-order model. The model is based on the following assumptions: the cantilever is described by the Euler–Bernoulli beam model, only the first flexural mode is considered, the perforation is treated as a small local material removal such that a first-order perturbation approximation is applicable, and the mode shape remains unchanged after perforation. Under these assumptions, the local reductions in stiffness and damping are represented by the corresponding weighting functions evaluated at $\xi_{0}$ and scaled by α. The model is intended to describe the spatial trend of the perforation effect. The limitation of this model is that it provides a simplified description of the perforation effect: it captures the spatial trend of stiffness loss and damping reduction, but does not resolve the three-dimensional fluid–structure interaction around the perforation.

(5) $$∆K\left(\xi_{0}\right)=\frac{EI}{L^{3}}{\left(\phi^{\prime \prime }\left(\xi_{0}\right)\right)}^{2}\alpha$$

(6) $$∆C\left(\xi_{0}\right)=c_{f}L{\left(\phi \left(\xi_{0}\right)\right)}^{2}\alpha$$

To evaluate the relative effect of perforation at different locations, the ratio of damping reduction to stiffness loss is defined as

(7) $$R\left(\xi_{0}\right)=\frac{∆C\left(\xi_{0}\right)}{∆K\left(\xi_{0}\right)}=\left(\frac{c_{f}L^{4}}{EI}\right){\left(\frac{\phi \left(\xi_{0}\right)}{\phi^{\prime \prime }\left(\xi_{0}\right)}\right)}^{2}$$

**Figure 3:**
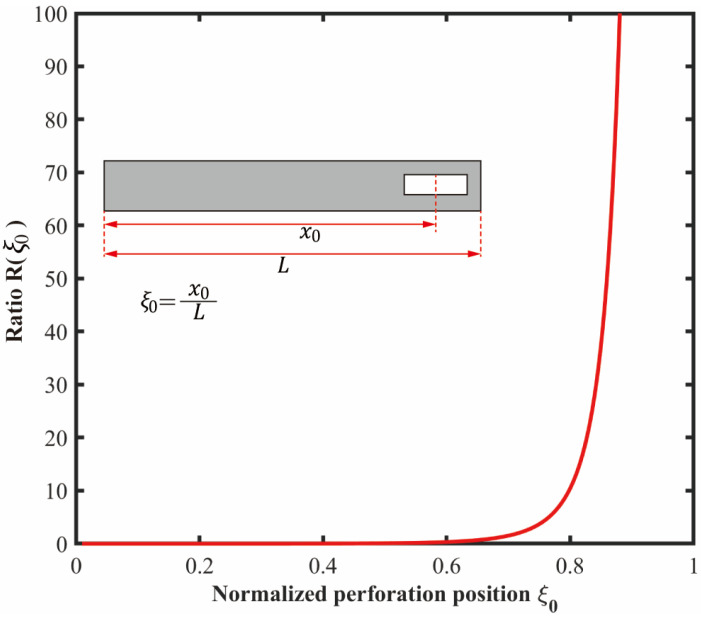
Ratio $(\phi (\xi_{0})/\phi^{\prime \prime }(\xi_{0}){)}^{2}$ as a function of normalized perforation position $\xi_{0}$.

The location dependence of $R(\xi_{0})$ is determined by the geometric term ${\left(\phi \left(\xi_{0}\right)/\phi^{\prime \prime }\left(\xi_{0}\right)\right)}^{2}$. Figure 3 shows the variation in this ratio along the beam. Near the clamped end, $R(\xi_{0})$ approaches zero because the modal displacement is small while the modal curvature remains large. In this region, perforation mainly reduces stiffness with limited reduction in damping. As $\xi_{0}$ approaches the free end, $R(\xi_{0})$ increases because the modal displacement increases while the modal curvature decreases. In the limit $\xi_{0}\to 1$, $\phi^{\prime \prime }(\xi_{0})\to 0$ due to the stress-free boundary condition, and $R(\xi_{0})$ becomes large. Thus, within the present model, perforation near the free end gives the largest ratio of damping reduction to stiffness loss. In this work, α and $\xi_{0}$ are introduced in the theoretical model to describe the general influence of perforation size and perforation position.

In the experiments, however, the perforation size is kept the same for the center-perforated cantilever (CPC) and FPC, and only the perforation position is changed for comparison.

### 2.4. Theoretical Prediction of Q-Factor via Perforation Design

Based on the reduced-order model in Equations (5) and (6), the effective stiffness $K_{p}\left(\xi_{0},\alpha \right)$, effective damping $C_{p}\left(\xi_{0},\alpha \right)$, and effective mass $M_{p}\left(\xi_{0},\alpha \right)$ for a perforated beam are written as

(8) $$K_{p}\left(\xi_{0},\alpha \right)=K_{solid}\left(1-\alpha \frac{{\left(\phi^{\prime \prime }\left(\xi_{0}\right)\right)}^{2}}{I_{K}}\right)$$

(9) $$C_{p}\left(\xi_{0},\alpha \right)=C_{solid}\left(1-\alpha \frac{{\left(\phi \left(\xi_{0}\right)\right)}^{2}}{I_{C}}\right)$$

(10) $$M_{p}\left(\xi_{0},\alpha \right)=M_{solid}\left(1-\alpha \frac{{\left(\phi \left(\xi_{0}\right)\right)}^{2}}{I_{M}}\right)$$

where $K_{solid}$, $C_{solid}$, and $M_{solid}$ are the effective stiffness, effective damping, and effective mass of the solid beam, and the mass integration constant is given by $I_{M}=\int_{0}^{1}\left(\phi (\xi \right){)}^{2}d\xi$. For the first order mode and the present normalization, $I_{M}=1$. For an equivalent single degree of freedom system, the Q-factor satisfies $Q\propto \frac{\sqrt{K_{eff}M_{eff}}}{C_{eff}}$. Thus, the Q-factor ratio of the perforated beam to the solid beam is

(11) $$\frac{Q_{p}}{Q_{solid}}=\frac{\sqrt{{(K}_{p}/K_{solid})(M_{p}/M_{solid})}}{C_{p}/C_{solid}}$$

Substituting Equations (8)–(10) into Equation (11) gives

(12) $$\frac{Q_{p}\left(\xi_{0},\alpha \right)}{Q_{solid}}=\frac{\sqrt{\left((1-\alpha \frac{{\left(\phi^{\prime \prime }\left(\xi_{0}\right)\right)}^{2}}{I_{K}}\right)\left(1-\alpha \frac{{\left(\phi \left(\xi_{0}\right)\right)}^{2}}{I_{M}}\right)}}{1-\alpha \frac{{\left(\phi \left(\xi_{0}\right)\right)}^{2}}{I_{C}}}$$

Since $I_{M}=I_{C}$ in the present model, substituting $I_{K}=12.362$ and $I_{C}=1$ into Equation (12) yields

(13) $$\frac{Q_{p}\left(\xi_{0},\alpha \right)}{Q_{solid}}=\sqrt{\frac{1-\alpha \frac{{\left(\phi^{\prime \prime }\left(\xi_{0}\right)\right)}^{2}}{12.362}}{1-\alpha {\left(\phi \left(\xi_{0}\right)\right)}^{2}}}$$

The resonant frequency is also affected by perforation through the changes in effective stiffness and effective mass. In the present reduced-order model, the normalized resonant frequency ratio is

(14) $$\frac{f_{r,p}}{f_{r,solid}}=\sqrt{\frac{K_{p}/K_{solid}}{M_{p}/M_{solid}}}$$

**Figure 4:**
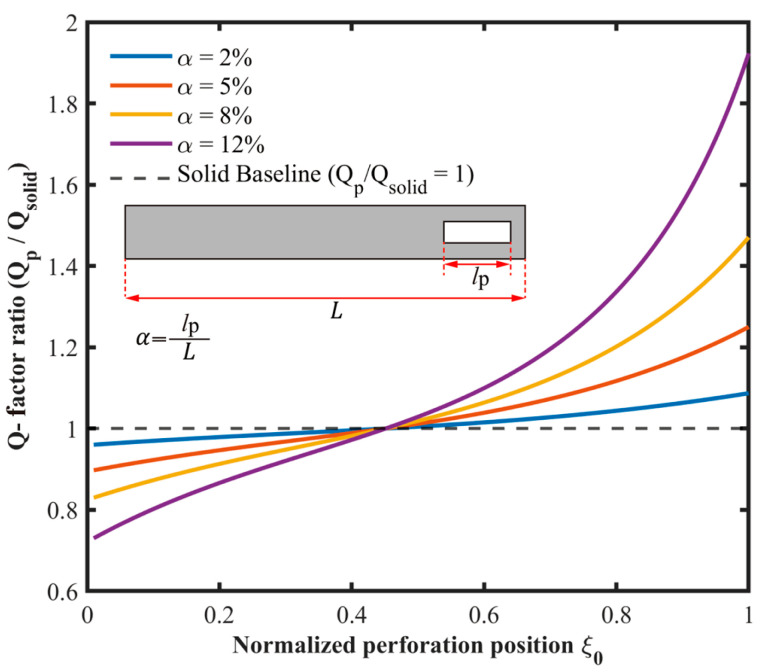
Q-factor ratio $Q_{p}/Q_{solid}$ versus normalized perforation position $\xi_{0}$ for different normalized perforation lengths α.

In the present model, both the resonant frequency ratio and the Q-factor ratio are determined by the modal weighting functions $\phi^{\prime \prime }\left(\xi_{0}\right)$ and $\phi \left(\xi_{0}\right)$. Hence, the frequency ratio exhibits a qualitatively similar dependence on perforation position as the Q-factor ratio, and a separate plot is omitted for brevity. This equivalence is specific to the reduced-order model used here and follows from the first mode assumption, the first-order perturbation approximation, and the unchanged mode shape assumption. The above expression links the perforation parameters to the Q-factor variation. Figure 4 shows $Q_{p}/Q_{solid}$ as a function of perforation position $\xi_{0}$ for different normalized perforation lengths α. For perforations near the clamped end, $Q_{p}/Q_{solid}<1$. The ratio exceeds 1 after the perforation position passes a threshold and then increases as the perforation moves toward the free end.

**Figure 5:**
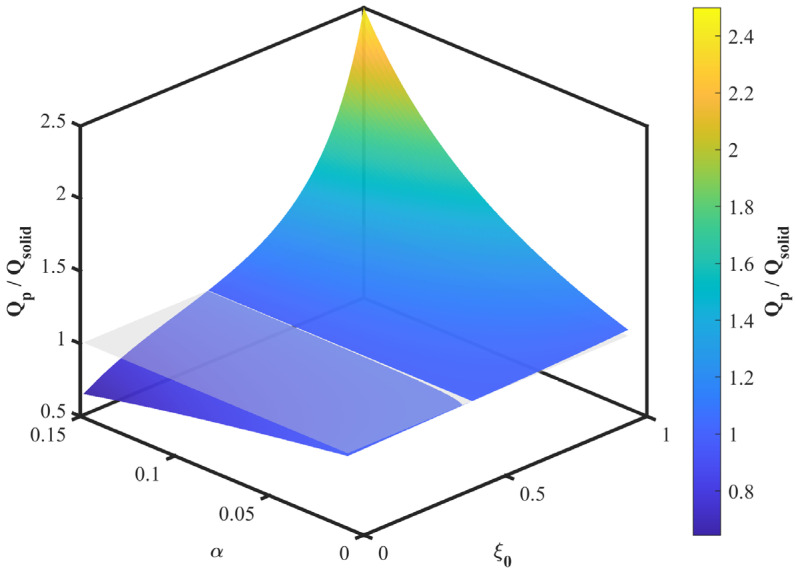
Surface plot of $Q_{p}/Q_{solid}$ as a function of normalized perforation position $\xi_{0}$ and normalized perforation length α.

Figure 5 shows $Q_{p}/Q_{solid}$ as a function of $\xi_{0}$ and α in a three-dimensional surface. The surface exhibits lower values near the clamped end and higher values near the free end. The maximum occurs at $\xi_{0}=1$, where $Q_{p}/Q_{solid}$ increases with α. In the present model, the spatial trend is mainly determined by the mode shape weighting functions. Fluid viscosity, fluid density, and beam dimensions mainly affect the absolute values of the dynamic parameters, but they do not change the predicted preferred perforation region within this reduced-order analysis.

These numerical results support the theoretical prediction that a perforation near the free end gives a larger damping reduction with a smaller stiffness penalty. As $\xi_{0}$ approaches 1, $\phi^{\prime \prime }\left(\xi_{0}\right)$ approaches 0, reducing the stiffness penalty, while $\phi \left(\xi_{0}\right)$ approaches its maximum, increasing the damping reduction.

## 3. Experimental Validation

### 3.1. Resonators Fabrication and Liquids Preparation

**Figure 6:**
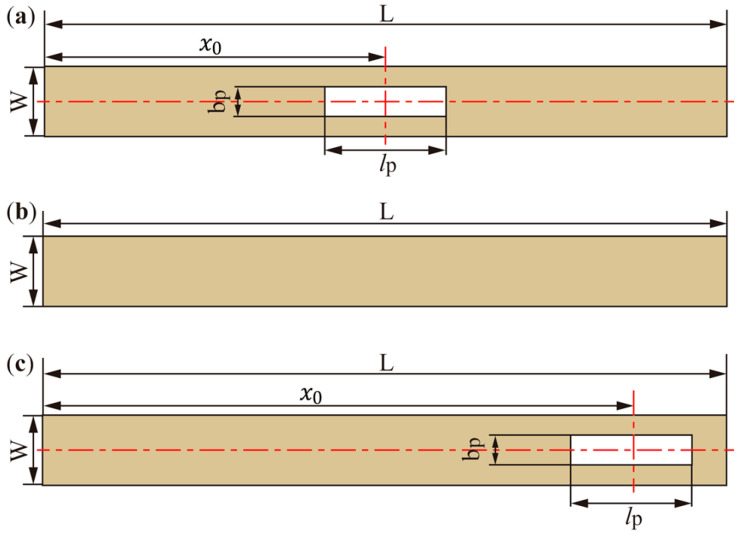
Geometries of the three cantilever resonators: (**a**) Center-perforated cantilever (CPC); (**b**) Non-perforated cantilever (NPC); (**c**) Free-end perforated cantilever (FPC). The CPC and FPC have the same perforation length $l_{p}$ and width $b_{p}$ but different perforation positions $x_{0}$. The perforation is a local rectangular through hole, and the beam length remains L. In (**a**,**c**), the horizontal red dotted line marks the common centerline of the beam and the perforation, and the vertical red dotted line marks the centerline of the perforation length.

To validate the theoretical analysis, three cantilever resonators are designed and fabricated: a center-perforated cantilever (CPC, Figure 6a), a non-perforated cantilever (NPC, Figure 6b), and a free-end perforated cantilever (FPC, Figure 6c). In the CPC and FPC, the perforation has the same size, and only the perforation position is different. Therefore, the experiments focus on the effect of perforation position under a fixed perforation size. The perforation is a local rectangular through hole. The prototypes are fabricated from brass by wire cutting, and the main geometric dimensions are verified before testing. The resonator geometries are illustrated in Figure 6, and the main material and structural parameters are listed in Table 1. Additionally, three liquid samples—denoted as G1, G2, and G3—are prepared by mixing glycerol and water in different proportions, all with an approximately constant density of 1220 kg/m^3^.

### 3.2. Experimental Approach and Results

**Figure 7:**
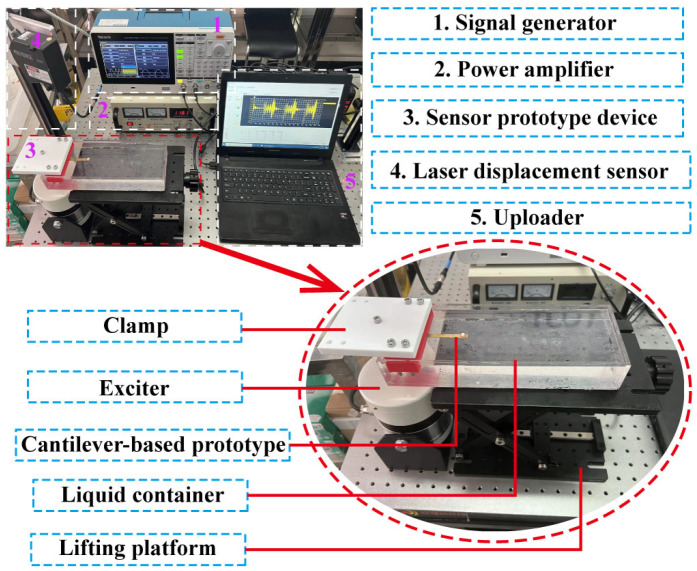
The experimental setup for measuring the resonant frequency and Q-factor of the cantilever resonators in liquids.

**Figure 8:**
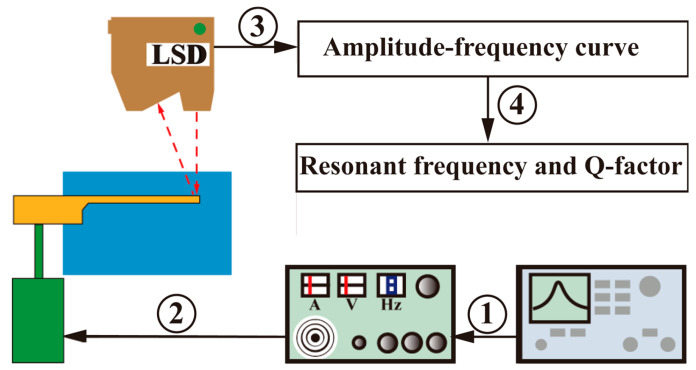
A schematic of the experimental procedure, including swept-frequency excitation, vibration measurement, and extraction of resonant frequency and Q-factor from the measured response.

An experimental setup is established to validate the theoretical model, as shown in Figure 7. The system consists of a signal generator (AFG3252C, Tektronix, Beaverton, OR, USA) and power amplifier (HEAS-50, Foeng, Nanjing, China) for signal excitation, an electromagnetic exciter (HEV-50, Foeng, Nanjing, China) to drive the cantilever resonator, a laser displacement sensor (LK-H150, Keyence, Osaka, Japan) for vibration measurement, and a data acquisition unit, clamp, liquid container, and lifting platform for sample positioning. The experimental approach is illustrated in Figure 8. Swept frequency signals generated by the signal generator are amplified and fed into the electromagnetic exciter, which drives the cantilever prototype. The resulting amplitude–frequency response is measured using the laser displacement sensor. The resonant frequency and Q-factor are then extracted from the response curve using the half-power bandwidth method.

**Figure 9:**
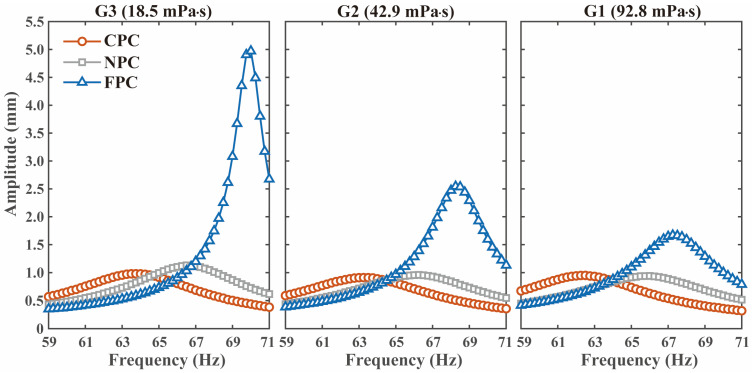
The measured amplitude–frequency curves of the CPC, NPC, and FPC in liquids G3, G2, and G1.

**Figure 10:**
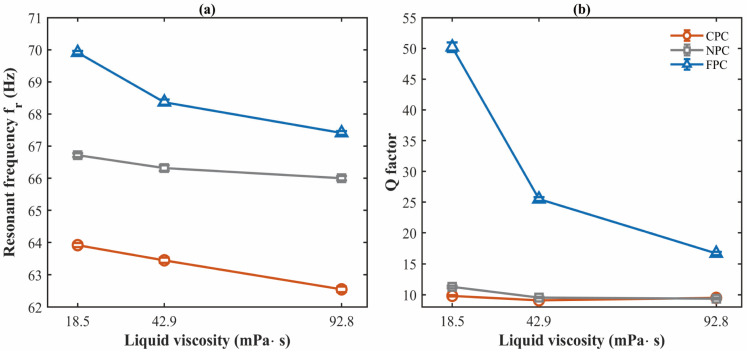
The extracted resonant frequency (**a**) and Q-factor (**b**) of the CPC, NPC, and FPC in liquids with viscosities of 18.5, 42.9, and 92.8 mPa·s. Error bars represent ±1 standard deviation from 10 independent measurements.

Experiments are conducted at a controlled temperature of 20 °C to minimize thermal drift. The measured amplitude–frequency curves are shown in Figure 9, and the extracted resonant frequency and Q-factor are summarized in Figure 10 and Table 2. For each liquid, 10 independent measurements are performed for each resonator. The values in Table 2 are reported as mean ± standard deviation, and the error bars in Figure 10 represent ±1 standard deviation.

Compared with the NPC, the FPC shows increases in both resonant frequency and Q-factor in all tested liquids. In the liquid with a viscosity of 18.5 mPa·s, the FPC reaches 69.910 ± 0.056 Hz and 50.20 ± 0.78, compared with 66.719 ± 0.062 Hz and 11.26 ± 0.20 for the NPC. In contrast, the CPC shows a lower resonant frequency than the NPC in all tested liquids and a lower Q-factor in two of the three liquids. For the resonant frequency, the coefficient of variation is below 0.14% for all tested cases. For the Q-factor, the coefficient of variation is below 2.2%, indicating good repeatability. The main sources of experimental uncertainty include clamp repeatability, laser spot alignment, excitation stability, liquid temperature fluctuation, liquid level positioning during immersion, and small fabrication deviations in the perforation geometry. Despite these uncertainties, the experimental results remain consistent with the theoretical prediction that a free-end perforation provides a larger damping reduction with a smaller stiffness penalty.

## 4. Conclusions

This study developed and experimentally validated a cantilever resonator with free-end perforations to suppress squeeze-film viscous damping introduced by liquid environments. A distributed-parameter model was established to quantify the spatial contributions of stiffness and damping along the cantilever. The analysis identified the free end as the optimal perforation location, where material removal reduces hydrodynamic drag without compromising stiffness. The experimental results, supported by rigorous repeatability testing, confirmed these predictions. The free-end perforated cantilever (FPC) achieved a 346.2 % increase in Q-factor and a 4.78 % increase in resonant frequency compared to the non-perforated cantilever (NPC), eliminating the need for complex higher-order mode excitation and the associated drive electronics that are common in previous approaches. These results provide a highly reliable design guideline for high-performance MEMS cantilever resonators in liquid applications.

## Figures and Tables

**Table 1:** Main material and structural parameters of the designed cantilever resonators.

Parameters	Values
Length L	65 mm
Width W	4 mm
Thickness H	0.8 mm
$\mathrm{Perforation}\;\mathrm{length}\;l_{p}$	2 mm
$\mathrm{Perforation}\;\mathrm{width}\;b_{p}$	1 mm
Poisson’s ratio	0.35
Mass density	8500 kg/m^3^
Elastic modulus	110 GPa

**Table 2:** The resonant frequency and Q-factor of the CPC, NPC, and FPC in liquids G3, G2, and G1. Values are mean ± standard deviation from 10 independent measurements.

Resonator	Liquid	Viscosity	Resonant Frequency	Q-Factor
FPC	G3	18.5	69.910 ± 0.056	50.20 ± 0.78
FPC	G2	42.9	68.369 ± 0.079	25.49 ± 0.30
FPC	G1	92.8	67.410 ± 0.057	16.68 ± 0.25
NPC	G3	18.5	66.719 ± 0.062	11.26 ± 0.20
NPC	G2	42.9	66.320 ± 0.085	9.56 ± 0.13
NPC	G1	92.8	66.000 ± 0.068	9.37 ± 0.16
CPC	G3	18.5	63.919 ± 0.065	9.82 ± 0.12
CPC	G2	42.9	63.450 ± 0.080	9.10 ± 0.19
CPC	G1	92.8	62.550 ± 0.086	9.49 ± 0.19

## Data Availability

All data used in the study are mentioned in the article.
